# Advancing Computational Toxicology by Interpretable Machine Learning

**DOI:** 10.1021/acs.est.3c00653

**Published:** 2023-05-24

**Authors:** Xuelian Jia, Tong Wang, Hao Zhu

**Affiliations:** † Department of Chemistry and Biochemistry, Rowan University, Glassboro, New Jersey 08028, United States

**Keywords:** Machine learning, Interpretable modeling, Computational toxicology, Risk assessment, Systems toxicology, Adverse outcome pathway

## Abstract

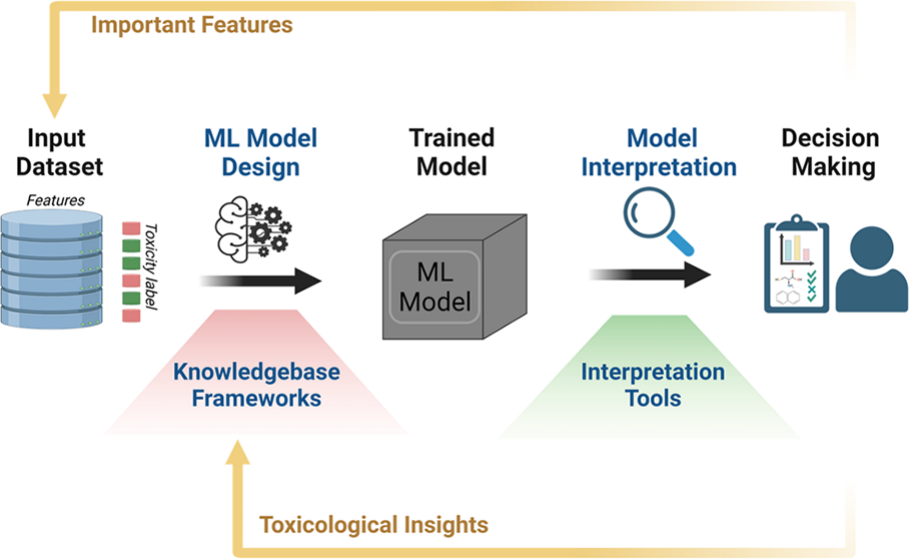

Chemical toxicity evaluations for drugs, consumer products, and environmental chemicals have a critical impact on human health. Traditional animal models to evaluate chemical toxicity are expensive, time-consuming, and often fail to detect toxicants in humans. Computational toxicology is a promising alternative approach that utilizes machine learning (ML) and deep learning (DL) techniques to predict the toxicity potentials of chemicals. Although the applications of ML- and DL-based computational models in chemical toxicity predictions are attractive, many toxicity models are “black boxes” in nature and difficult to interpret by toxicologists, which hampers the chemical risk assessments using these models. The recent progress of interpretable ML (IML) in the computer science field meets this urgent need to unveil the underlying toxicity mechanisms and elucidate the domain knowledge of toxicity models. In this review, we focused on the applications of IML in computational toxicology, including toxicity feature data, model interpretation methods, use of knowledge base frameworks in IML development, and recent applications. The challenges and future directions of IML modeling in toxicology are also discussed. We hope this review can encourage efforts in developing interpretable models with new IML algorithms that can assist new chemical assessments by illustrating toxicity mechanisms in humans.

## 1 Introduction

Chemical risk assessments and safety testing are important for the early identification of hazardous chemicals in multiple industry sectors.^1−3^ For example, in the drug development procedure, toxicity evaluation in the early stage can reduce the attrition rate and late failure, which significantly reduce the cost of developing a new drug.^4,5^ Traditional toxicity evaluations for pharmaceuticals, xenobiotics, and environmental chemicals often involve the use of toxicological tests conducted in animal models, which are expensive, time-consuming, and raise concerns about animal welfare. The rapidly increasing number of chemicals in medical, industrial, and agricultural fields has made it impractical to use animal models for evaluating tens of thousands of new chemicals.^6,7^ As an alternative strategy, computational toxicology using machine learning (ML) techniques has shown promise for chemical toxicity evaluations because it can quickly predict the toxicity of a large number of new compounds in the risk assessment process and prioritize potentially hazardous compounds for experimental testing.^8^ In the National Research Council (NRC) 2007 report *Toxicity Testing in the 21st Century: A Vison and a Strategy*, the development of computational techniques for risk assessment was emphasized.^9,10^ In 2016, the Frank R. Lautenberg Chemical Safety for the 21st Century Act (LCSA) was approved to advance chemical risk assessment. The LCSA called for computational approaches and strategies for safety evaluation to reduce or replace the use of vertebrate animals while providing evidence to support regulatory decisions.^11^ In chemical industries (e.g., consumer products), the use of computational models for chemical toxicity assessments is also important for decision-making during product development.^12^

In the past decade, the development of new experimental protocols, especially high-throughput screening (HTS) assays, and the progress of combinatorial chemistry has generated toxicity data for millions of compounds.^8,9^ With the development of advanced ML and deep learning (DL) algorithms, computational modeling can use massive toxicity data for more accurate chemical toxicity predictions. Some DL models have been shown to match or even outperform other ML algorithms on prediction accuracy.^13−16^ However, a common limitation of complex ML models, especially DL models using neural network architecture, is their “black box” nature, which means their inner working mechanisms cannot be easily understood by users.^17^ There is an increasing demand for developing strategies to help toxicologists understand the model and how the predictions are made. The development of interpretable ML (IML) is an effective approach to mitigate the lack of interpretability underlying a trained model to reveal underlying toxicity mechanisms and to augment decision-making.

ML models are being ubiquitously used in daily lives (e.g., hiring, advertising, and music recommendations)^18−20^ and health-related fields (e.g., healthcare, risk assessment, and drug discovery).^21−23^ However, without an understanding of working mechanisms, black box models can lead to mistrust of the results.^24^ In health-related fields, black box models can negatively affect human health, racial bias, and safety.^25−28^ For example, Obermeyer et al. uncovered a racial bias issue in a widely used model for predicting health needs.^26^ Some pollution models incorrectly predicted highly polluted air as nonhazardous to humans, due to the unknown working mechanism of the models.^27^ Such negative consequences caused by black-box ML models can be avoided by developing IML with increased transparency and interpretability.^29,30^ Lundberg et al. reported the use of IML for the prevention of hypoxemia during surgery, which can provide explanations of the risk factors in real-time and increase the anesthesiologists’ anticipation of hypoxemia events by 15%.^31^ Recent applications of IML in the healthcare field showed IML’s potential in detecting bias and ensuring the interpretability of the model while maintaining accuracy.^32−35^

IML can perform robust model validations to avoid making wrong decisions learned from biased training data and make the underlying decision-making understandable.^36−39^ Ideally, besides the predictions made by a model, the knowledge about chemical toxicants in the training data can help toxicologists better evaluate the trained model and make decisions on new compounds, i.e., determining which environmental chemicals and drugs are of the greatest potential concern to human health.^40^ Because different scientific communities use ML for different prediction tasks, there is no universal definition of IML.^36^ Regarding the chemical toxicity assessments, the desired IML models need to fulfill the following criteria:

- Models should be constructed by explicit/understandable architectures.
- Users can understand how the model reached a specific prediction.
- Models can provide toxicity knowledge insights to support decision-making.

Recent efforts to develop IML and facilitate its application in toxicology are discussed in this review. We first overview feature data of chemicals that can be used for toxicity model development. Then, examples of computational algorithms and their specific interpretation methods are presented, followed by strategies for explaining black box models. The use of biological and toxicological knowledge in guiding the design of IML models is then discussed. Finally, we conclude with potential challenges in the practical applications of IML in toxicology.

## 2 Feature Data in Computational Toxicology Modeling

ML is the technique to build predictive models by learning from input feature data using computational algorithms.^41,42^ A typical procedure to develop a predictive model includes data collection and curation, model building, and model validation. Different types of data present different features and different levels of interpretability for users.^36^ Unlike raw data (such as pixel units in an image),^29^ most of the data used in toxicity modeling reflect the properties/activities of chemicals, which possess the ability to be interpreted during the modeling process and/or after the model has been developed. Therefore, the training data consisting of different feature types in toxicological modeling is the base of IML.

### 2.1 Structural/Chemical Properties

**Figure 1:**
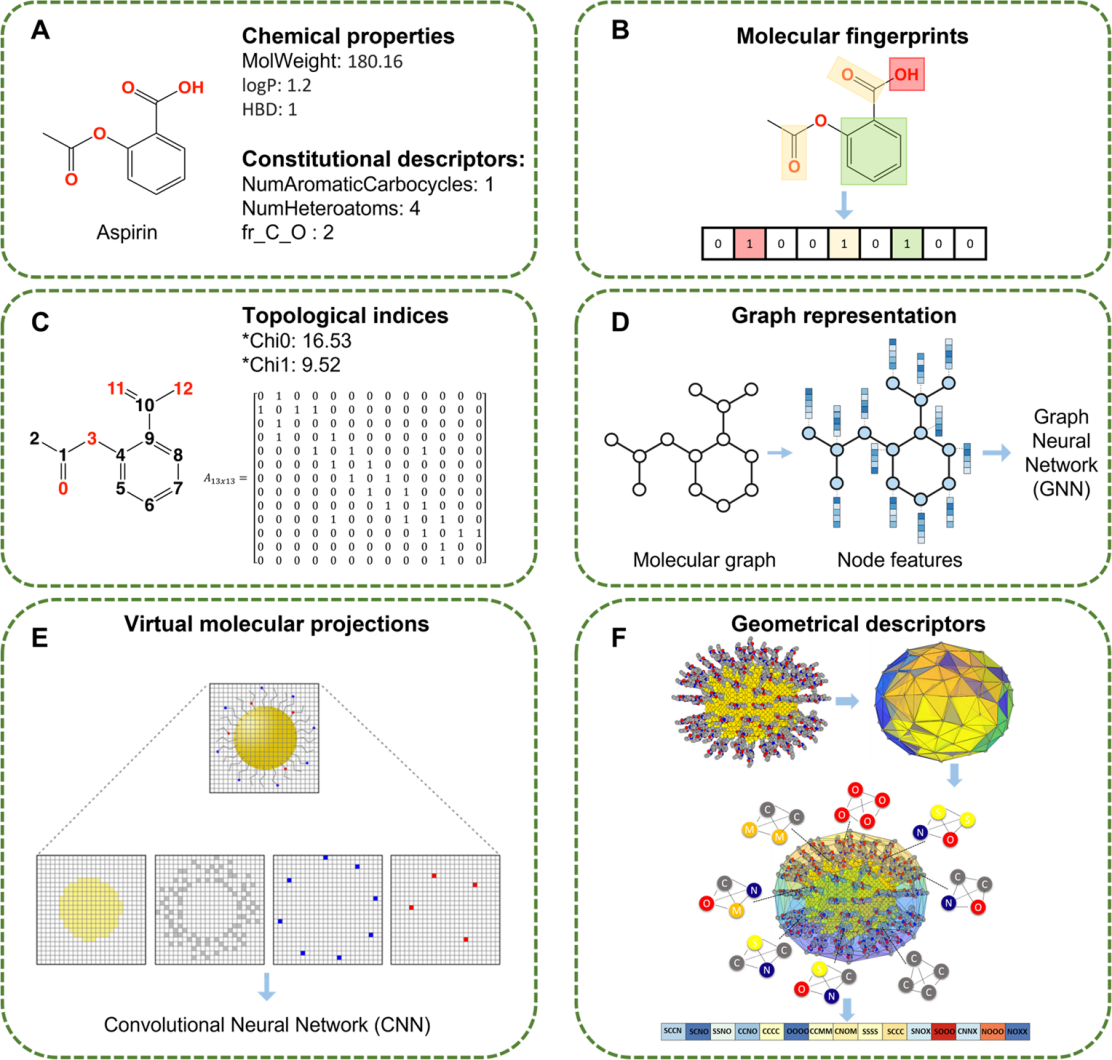
Examples of different types of chemical descriptors. (A) Chemical properties and constitutional descriptors. (B) Molecular fingerprints as a vector of bits that denotes the presence “1” or absence “0” of a specific structural feature. (C) Topological indices as global features that derive information from the adjacency matrix of the molecular graph of a chemical (*calculated on the basis of eqs 1, 9, 10, and 1, 11, and 12 of ref (56)). (D) Graph representation of a molecule including node features (e.g., atom type and aromaticity), adjacency matrix, and edge features, which can be used as inputs to a Graph Neural Network (GNN). (E) Virtual molecular projections of nanoparticles (Reprinted with permission from ref (51). Copyright 2020 American Chemical Society). (F) Geometrical surface descriptors of nanoparticles (Reprinted with permission from ref (52). Copyright 2019 Royal Society of Chemistry).

For toxicity modeling, the most intuitive data to be used is the chemical structure information. To make it machine-readable during modeling, chemical structures need to be transformed into vectors of numerical or binary values.^36^ Quantitative structure–activity relationship (QSAR), a statistical approach that correlates a compound’s chemical structural or physicochemical properties to its activities, has been used traditionally for chemical toxicity modeling.^43^ The molecule structures were normally transferred into molecular descriptors at the beginning of a modeling procedure. The calculated molecular descriptors can represent local or global salient characteristics of the structures (Figure 1). Major classes of descriptors include (a) physicochemical descriptors (such as molecular weight, lipophilicity, etc.; Figure 1A) representing properties determining the absorption and distribution of chemicals in the body; (b) fingerprints, which are binary bits representing the presence “1” or absence “0” of substructures and molecular features of interest (Figure 1B); (c) constitutional descriptors representing the counts for corresponding atoms, bonds, and functional groups (Figure 1A); (d) geometrical descriptors capturing the three-dimensional structure features, such as the molecular size and shape (Figure 1E); and (e) atom distributions and topological indices representing the connectivity of atoms in the molecules (Figure 1C).^44,45^ The structure information on chemicals can be stored in various formats, including linear representations such as SMILES (Simplified Molecular Input Line Entry Specification) and InChI (the IUPAC International Chemical Identifier) and connection table-based file formats such as SDF (Structure Data Format).^46,47^ These chemical structure data can be accessed from chemical data-sharing repositories, such as PubChem and ChEMBL (Table 1), and can be further processed by cheminformatics tools (e.g., RDKit, http://www.rdkit.org/, accessed January 2023) to generate descriptors for toxicological modeling.

**Table 1:** Publicly Available Big Data Repositories for Toxicology Modeling^a^

database	data type	description
PubChem^69,70^	chemical molecules, biological activities	Over 110 million compounds and over 1.5 million bioassays related to toxicity, genomics, and literature data
ChEMBL^85^	chemical molecules with druglike properties, bioactivity, and genomic data	Contains a total of >15 million bioactivity measurements for 1.8 million compounds and pharmacokinetic measurements for 85 drugs
ToxCast^63,64^	toxicity-related *in vitro* assays	Phase I: 309 compounds (mostly pesticides) tested in ∼500 HTS assays in human primary cells, cell lines, and rat primary hepatocytes
		Phase II: additional 776 compounds, including failed pharmaceuticals tested in ∼700 HTS assays
Tox 21^61,62^	toxicity-related *in vitro* assays	Phase I: ∼2800 compounds tested in ∼75 nuclear receptor and stress response pathway assays
		Phase II: expand to ∼10 000 chemicals including all marketed pharmaceuticals
Integrated Chemical Environment (ICE)^86−88^	curated *in vivo* and *in vitro* toxicity data	Provides high-quality curated *in vivo* and *in vitro* test data, reference chemical lists, and computational tools for chemical characterization and toxicity prediction. Curated HTS (cHTS) data in ICE are curated to bolster data confidence and are annotated to mechanistic targets
Gene Expression Omnibus (GEO)^82,83^	genomics data	Public repository that archives and freely distributes genomics data submitted by the research community and stores approximately a billion gene expression measurements from over 100 organisms
Open TG-GATEs^77^	*in vivo* and *in vitro* toxicogenomic data	Microarray gene expression data of 170 compounds tested in primary rat and human hepatocytes and *in vivo* rat liver and kidney with multiple treatment durations at three different doses
DrugMatrix^78^	*in vivo* and *in vitro* toxicogenomic data	Microarray gene expression data of ∼600 different compounds in rat hepatocytes and up to eight rat tissues with different durations of exposure
LINCS L1000^81^	*in vitro* toxicogenomic data	High-throughput measurements of 978 landmark gene set were used to infer the whole genome-wide expression (11 350 inferred genes), with ∼20,000 compounds tested at a variety of time points, doses, and in nine core human cell lines
Immunological Genome Project (ImmGen)^89^	genes and their networks in the immune system	Largest publicly available compendium of genome-wide transcriptional expression profiles for ∼250 distinct immunological cell states in mice

a LINCS, NIH Library of Integrated Network-Based Cellular Signatures; Open TG-GATES, Open Toxicogenomics Project-Genomics-Assisted Toxicity Evaluation System.

Besides structural/chemical descriptors, chemical molecules can be treated as graphs, where graph embedding techniques are applied to generate feature vectors for modeling. During the graph embedding process, chemical molecules are first represented as undirected graphs with atoms as nodes and edges as bonds (Figure 1D), and then, embedding techniques are introduced to obtain a spatial graph matrix for each atom.^48−50^ These embeddings are used as features to train a model. The chemical molecules can also be transformed to image-like data. For example, in a nanotoxicity study, nanoparticle structures were transformed into “virtual molecular projections” (Figure 1E), which are multidimensional digital data representing the components of a nanoparticle structure without losing critical structure information.^51^ The atomic coordinates of a virtual nanoparticle are projected onto a 2D space on the basis of the atom type and coordinates in 3D space. These projections were then used as inputs to predict the properties and activities of nanoparticles using an image processing convolutional neural network (CNN). Structure annotation techniques, such as Delaunay tessellation, which decomposes the surface of nanostructures into tetrahedra, have been developed to generate nanodescriptors that simulate surface chemistry and properties of complex nanoparticle structures (Figure 1F).^52,53^ Overall, structure-based modeling, such as QSAR, is reliable in predicting some pharmacokinetic properties and *in vitro* assay responses with simple mechanisms for new compounds.^54,55^ However, for complex toxicity endpoints (e.g., carcinogenicity and hepatotoxicity), the use of only structural information and chemical properties for modeling (i.e., QSAR) is error-prone, particularly when compounds with similar structures or chemical properties exhibit dissimilar toxicities.^5^

### 2.2 Pathway-Based Toxicity Data

In the past decade, the development of automatic experimental screening technology has significantly enhanced the efficiency of *in vitro* biomolecular or cell-based assays, thereby resulting in the HTS technique capable of screening thousands to millions of compounds.^57,58^ The adverse outcome pathway (AOP) is a conceptual framework that links chemical-induced responses at the molecular, cellular, and organ levels to adverse outcomes at the organism level. Mechanism-based assay outcomes can be used within an AOP pathway to systematically assess whether a compound is likely to induce the target adverse outcome.^7,59^ The chemical responses obtained from target-specific, mechanism-oriented *in vitro* assays in HTS projects like ToxCast and Tox21^60−63^ keep growing and have contributed to the current toxicity big data (Table 1). Using a ToxCast/Tox21 assay, compounds were tested in multiple concentrations to generate concentration–response curves defining compound activity.^62,64^ Then, statistical analysis was performed to define mechanistic outcomes for tested compounds, such as receptor binding, inhibition, and activation representing key events of a toxicity pathway. The outcomes can be used as biological descriptors of chemicals, which can be further combined with molecular descriptors to improve the ML models.^65−67^ Moreover, quantitative outcomes from the concentration–response curves of active chemicals, such as half-maximal response concentration (AC_50_) or lowest effective concentration (LEC), can be used for the extrapolation of *in vivo* equivalent dose and prediction of toxicity potentials.^68^ In parallel with the progress of various HTS projects, several data-sharing projects were also developed in the past decade (Table 1). For example, PubChem is a public repository for over 110 million chemicals and their associated bioactivities.^69,70^ The tremendous amount of PubChem bioassay data that are updated daily constitutes a publicly accessible big data resource for compounds with a variety of target response information, which can also be used in toxicity ML and IML modeling studies.

### 2.3 Toxicogenomic Data

Cellular or organismal responses to chemical compounds are being measured at different levels. Genome-wide transcriptomic data enables the assessment of alterations in gene expression profiles induced by chemicals. The rapid increase of genomic-sequence data and associated gene annotations (e.g., gene ontology) also accelerate the application of gene-expression modeling to understand the toxicity mechanism of toxicants.^71,72^ Toxicogenomic data generated using these techniques provide extra valuable information in the chemical toxicity modeling process.^73−76^Table 1 also includes several toxicogenomic data repositories that store gene expression data of animals, human primary cells, and cell lines with/without exposure to drugs, industrial and environmental chemicals, etc. For example, Open TG-GATEs and DrugMatrix conducted short-term repeat-dose rat studies to obtain gene expression profiles coupling with histopathology measurements to enable a better understanding of chemical effects in rats.^77−79^ Meanwhile, results from rat and human hepatocytes for the same set of chemicals allowed the identification of similarities and relationships between the *in vitro* and *in vivo* systems.^80^ The L1000 project, as the next generation Connectivity Map, has developed a low-cost and high-throughput transcriptomic assay, which uses measurements of the 978 “landmark” genes to infer the expression levels of 81% of nonmeasured transcripts.^81^ It generates transcriptomic profiles in multiple human-derived cell lines for around 20 000 chemicals. Toxicogenomic data generated from the above projects are deposited in the National Institutes of Health’s Gene Expression Omnibus (GEO) database. GEO is an international public repository that archives and freely distributes microarray, next generation sequencing, and other forms of high-throughput functional genomics data submitted by the research community.^82,83^ In addition to gene expression data, some databases also provide associations between chemicals, protein/gene targets, and disease that can aid in the mechanistic modeling of chemical-induced adverse outcomes. For example, Comparative Toxicogenomics Database (CTD) is a publicly available database of manually curated toxicogenomic information extracted from literature (Table 2).^84^ It provides information for chemical–gene/protein interactions, chemical–disease associations, and gene–disease relationships, which can be integrated with pathway and functional data to facilitate the development of hypotheses about how environmental exposures influence human health.

**Table 2:** Knowledge Resources for Toxicological Modeling

resource type	name	description
AOP database	AOP-wiki (https://aopwiki.org, accessed January 2023)	One component of a larger OECD-sponsored AOP knowledge base (AOP-KB) effort, central web-based tool for disseminating and reviewing AOP knowledge; currently features more than 400 AOPs.
	AOP4EUpest^186^	A resource for annotated pesticides-biological events involved in AOPs.
gene (sets) annotation	Gene Ontology^178,179^	A comprehensive resource for computable knowledge of gene products comprised of gene ontology terms for many kinds of biological functions, involved pathways, and relationships between them.
	Molecular Signatures Database (MSigDB)^187,188^	Resource of ∼32 000 annotated gene sets for use with Gene Set Enrichment Analysis, including human and mouse collections.
pathway database	Kyoto Encyclopedia of Genes and Genomes (KEGG)^189,190^	KEGG has a collection of databases dealing with genomic information, biological pathway, diseases, drugs, and chemical substances. PATHWAY database contains pathway maps for the molecular systems in both normal and perturbed states.
	REACTOME^180^	A curated knowledge base of biological pathways in a hierarchical structure. It provides molecular details of biological processes as an ordered network of molecular transformations.
	Wikipathways^191^	A database of biological pathways collected and curated by the research community.
	Pathway commons^192^	Database that integrates data from public databases and contains over 5700 pathways and 2 million interactions.
toxicogenomics knowledge base	Comparative Toxicogenomics Database (CTD)^84^	Includes more than 30.5 million toxicogenomic connections relating chemical–gene/protein interaction, chemical–disease, and gene–disease relationships; gene ontology annotations; and pathways modules.
	Chemical Effects in Biological Systems (CEBS)^177,193^	Combines molecular expression data from transcriptomics, proteomics, metabonomics, and conventional toxicology with metabolic and toxicological pathway and gene regulatory network information relevant to environmental toxicology and human disease.
network analysis and visualization	Gephi^194^	An open-source visualization and exploration platform for all kinds of networks, complex systems, and graphs.
	Cytoscape^195^	Software platform for visualizing molecular interaction networks and biological pathways to integrate these networks with annotations, gene expression profiles, and other state data.

## 3 ML Approaches and Model-Specific Interpretation Methods for IML

**Figure 2:**
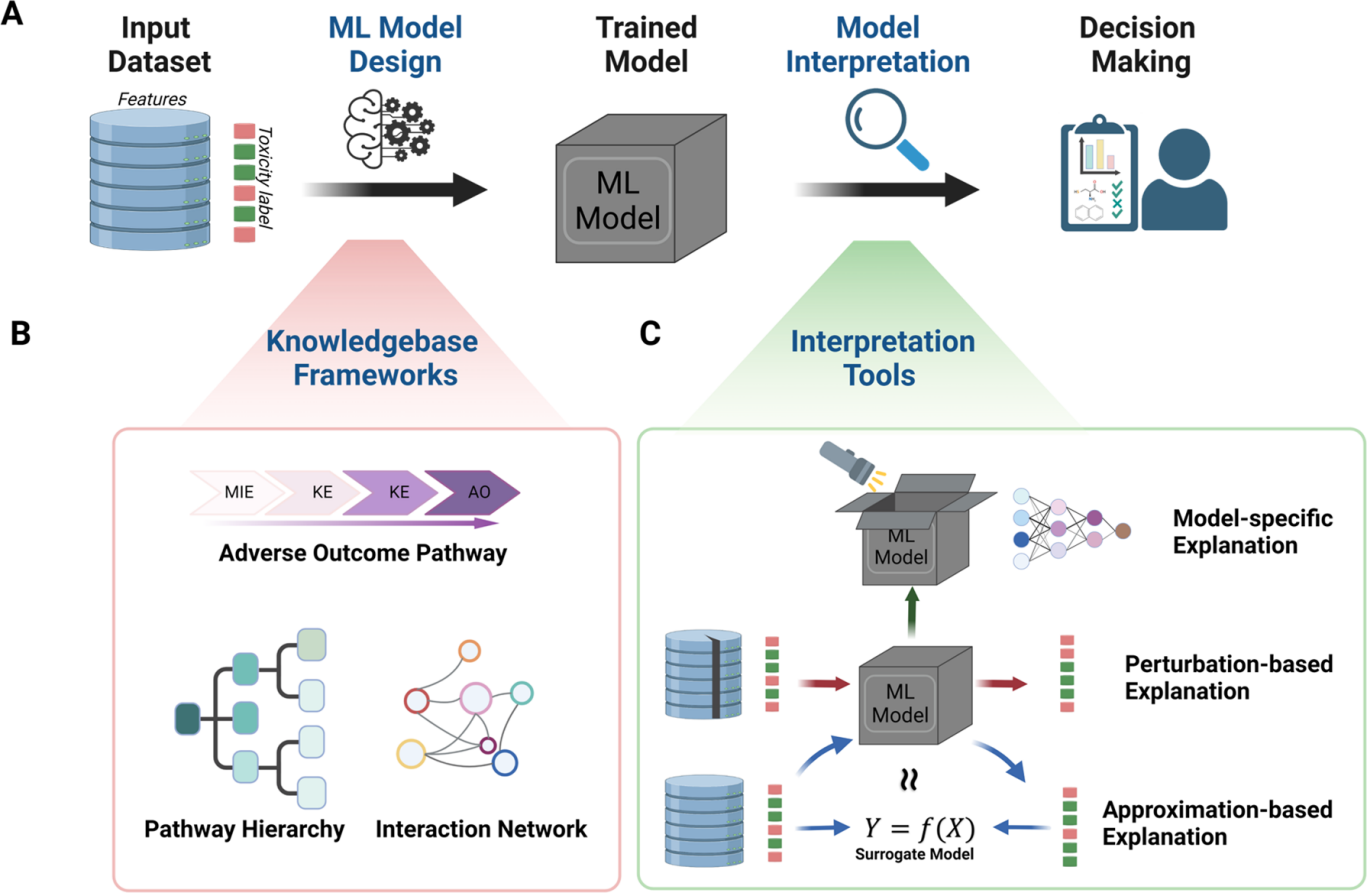
Strategies for the development of IML models for chemical toxicity. (A) Workflow for toxicity modeling using ML approaches where strategies can be applied before and after model training to improve intrinsic and posthoc interpretability, respectively. (B) Toxicological knowledgebase frameworks can be used to design models that are intrinsically interpretable. MIE, molecular initiating event; KE: key event; AO, adverse outcome. (C) With a trained model, interpretation tools can be applied to explain the trained model, examine important features, and support decision making.

The interpretability of ML models can be classified as intrinsic interpretability and posthoc interpretability^40,90,91^ and can be achieved before and after model training (Figure 2A). Intrinsic interpretability is achieved by constructing self-explanatory models (e.g., using toxicological knowledge base frameworks) (Figure 2B), which incorporate interpretability directly into the model structures.^90^ Understanding the inner logic of a ML algorithm is important for troubleshooting during model training. Posthoc interpretability is achieved after obtaining a trained model (Figure 2C). The goal of posthoc method is to understand the model predictions on the basis of the training data.^92^ This section overviews examples of important ML algorithms and algorithm-specific techniques for interpreting the derived ML models.

### 3.1 Classic ML Approaches

**Figure 3:**
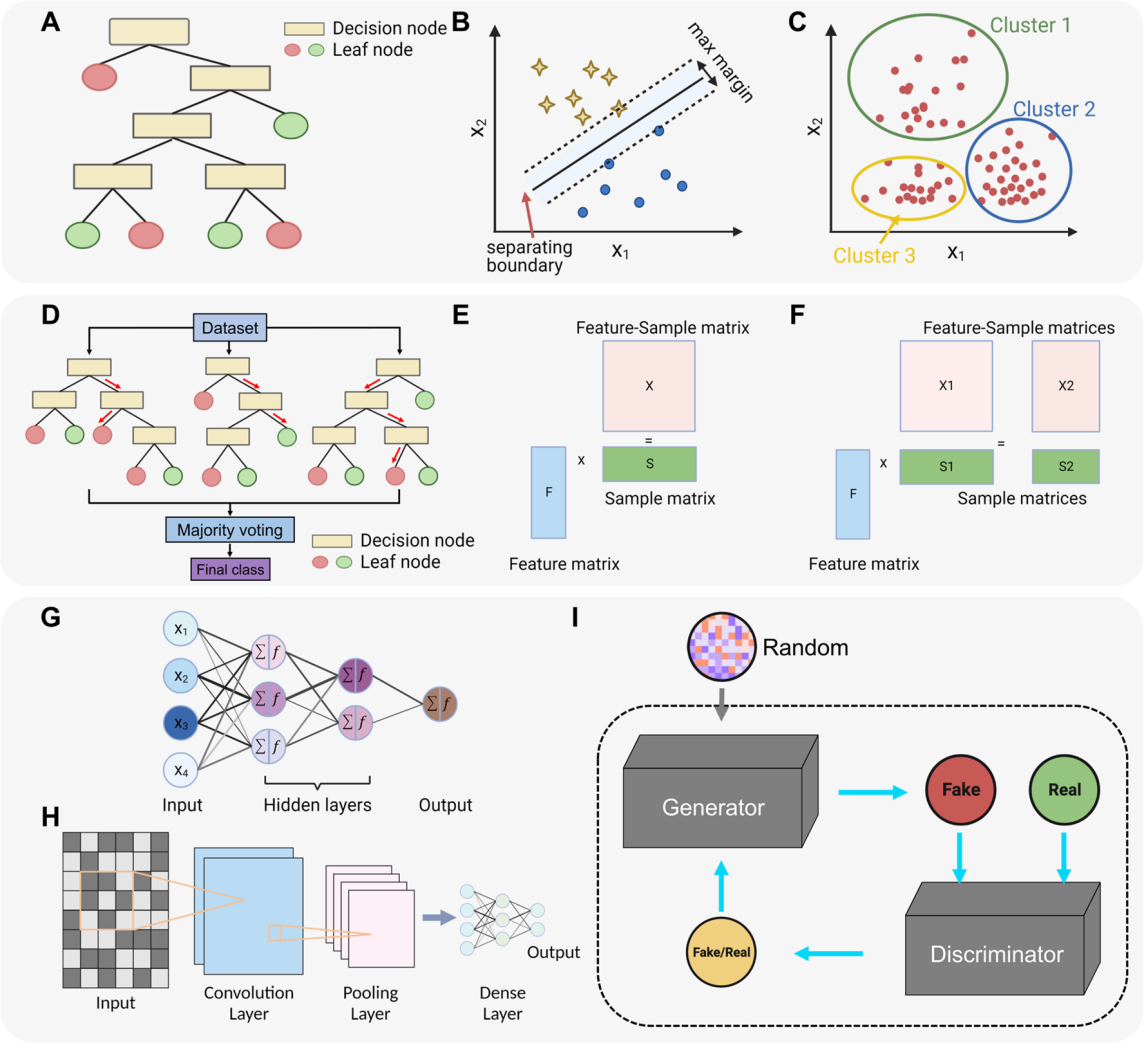
Examples of ML approaches: (A) decision tree, (B) support vector machine, (C) clustering, (D) random forest, (E) matrix factorization, (F) group factor analysis, (G) deep neural network, (H) convolutional neural network, (I) generative adversarial network.

ML includes three basic branches: supervised learning that aims to learn a mapping from features to labels (toxicity) on the basis of labeled training samples, unsupervised learning that aims to find patterns from unlabeled samples, and semisupervised learning that combines labeled samples with unlabeled samples during training.^93−95^ Most classic supervised learning algorithms are well studied and interpretable for humans, such as linear regression, decision rule, and decision tree.^91^ Linear regression models predict the target label as a weighted sum of feature data. The linearity of the learned relationship is easy to understand. For example, as a model of lipophilicity, logP is predicted using chemical structure or properties as regressors (e.g., functional groups, molecular volumes, and molecular weights).^96,97^ Decision rules are also interpretable models that follow a general IF–THEN structure: IF the conditions are met, THEN the model makes a certain prediction. The conditions are built from interpretable features where pairs of conditions can be combined with AND/OR.^36,91^ Decision trees are graphs to represent multiple true/false questions in a tree structure, where internal nodes represent tests on features to split the samples, edges represent the split decisions, and leaf nodes represent corresponding class labels (Figure 3A).^93^ Predictions for new chemicals are made by following a decision path from the root to the leaf of a developed decision tree model. In a modeling study of oral toxicity, a decision tree was constructed with 33 questions on the basis of structure, biochemistry, and physiological chemistry information.^98^ Each answer leads to another question and eventually ends with a final classification into one of three classes reflecting low, moderate, or serious toxicity. Support vector machine (SVM) is an algorithm that aims to find the hyperplane that best separates samples, such as chemicals, by their labels when the samples are placed in a high-dimensional feature space (Figure 3B). SVM can be trained to learn either linear or nonlinear relationships between features and labels. When dealing with nonlinear relationships, SVM projects the original data into a higher-dimension space where it can be separated by a linear hyperplane using the “kernel trick,” thereby making it less interpretable.^99,100^

Most of the above algorithms are interpretable since their structures and inner working mechanisms are transparent.^90^ Besides that, they are also interpreted on the modular level after model training, i.e., understanding the effects of training data and model parameters on predictions. For example, the weights of a linear model can be described as reflecting the strengths of relationships between features and target toxicity.^17^ The positive weight of a feature means this feature contributes by increasing the model’s output and vice versa.^36^ In a decision tree model, the importance of a feature can be computed by going through the splits where the feature was used and measuring the increase of accuracy compared with the parent node.^90,91^ By extracting the coefficient weights that define the hyperplane in a linear SVM model, toxicologists can interpret the features (molecular patterns) that were assigned a higher absolute weight as having stronger impacts on toxicity prediction.^101,102^ The interpretation of nonlinear SVM is relatively complex and should be based on the specific kernel used for transforming the data.^101,103,104^

It should be noted that when the size and complexity of a ML model increase, the model will become less interpretable.^36^ For example, a decision tree can be hard to interpret if it has a large width and depth. As an ensemble learning approach, random forest (RF) combines many decision trees to generate one prediction (Figure 3D) and is generally less interpretable.^105^ To resolve this issue, approaches estimating feature importance and contributions for RF models have been developed and used in toxicity QSAR modeling, which can identify chemical substructures or features expected to be responsible for toxicity.^106−109^ For example, Yu et al. proposed a tree-based RF feature importance and feature interaction network analysis to interpret the developed RF models for immune response and the lung burden of nanoparticles.^109^ In this study, multiple-indicator feature importance analysis (e.g., predicted label change, node purity increase, etc.) was used to identify important features, and feature interaction networks were built to explore the interactions among multiple features. The modeling approach selection should depend on the complexity of the problem, specifically the relationship between input features and target toxicity labels. Linear regression or linear SVM models will not be applicable when the relationship between input features and toxicity is not straightforward. During the interpretation of feature importance, correlated features may cause issues where weights are split between them and feature importance is underestimated. A possible solution is to apply feature selection to remove redundant or irrelevant features, which can reduce the model’s complexity and increase interpretability.^103,110−112^

### 3.2 DL Approaches

The advancement of computational infrastructure stimulated the applications of advanced ML algorithms to address the challenge of the explosive growth of toxicity data. DL is a part of the ML family based on artificial neural networks (ANN) with representation learning. DL algorithms are being applied widely in fields, including voice and image recognition, language processing, and clinic studies, because of their good predictive performance in modeling complex systems.^113−115^ The structure of an ANN mimics the interlinked neurons in the brain, where a set of input nodes connects to a second set of nodes called the “hidden” layer and then eventually to an output layer.^116^ A weight is associated with each of these connections between nodes, and there may be more than one hidden layer to construct a “deep” neural network (DNN) (Figure 3G). Other DL algorithms, such as CNN and adversarial learning, were designed for specific tasks. Inspired by the biological organization of the animal visual cortex, CNNs were constructed to learn spatial patterns or feature representation from input raw data (e.g., pixels from an image) (Figure 3H) which makes them ideal for image and speech applications.^117,118^ In a generative adversarial network (GAN), two DL models are trained during contesting with each other, the generative network generates synthetic data, and the discriminative network distinguishes synthetic data from true data distributions (Figure 3I), which is ideal for the generation of new data with similar statistics as the training set.^119^ A recent study developed a GAN-based modeling framework (Tox-GAN) that learned from existing animal transcriptomic profiles to generate new transcriptomic profiles on the basis of chemical structures, doses, and treatment durations.^73^ Tox-GAN can generate transcriptomic profiles without animal testing, which facilitates an understanding of toxicity mechanisms of new compounds and enhances the biomarker development in predictive toxicology.

Since the internal structures and underlying working mechanisms are less interpretable, DNN models are black-box in nature compared with classic ML models. To make predictions with a DNN model, input data pass through many layers of multiplications with the learned weights and through transformations by activation functions that can be nonlinear.^91^ This process may involve millions of weight parameters depending on the architecture of the DNN, thereby making it difficult to understand the meanings of inner neurons and weights and how the predictions are made. To resolve this issue, methods for interpreting DNN models have been developed and can be classified as three major categories. Backpropagation-based methods calculate the gradient of an output with respect to the inputs to derive the contributions of features.^90,120−123^ Connection weight-based methods track the magnitudes and directions of weights between neurons to identify individual and interacting effects of input variables on the outputs. It enables the estimations of feature importance when summing all connection weights.^124^ Investigation of neuron representations looks at the hidden neuron representations to provide explanations. For example, in CNN, visualization of the inner neurons' output can show the encoded meanings of the original image.^90,125,126^ In a DNN model for toxicity prediction using chemical structure data, Mayr et al. visualized the fragments represented in hidden neurons of different layers and found high correlations between neuron representations and toxicophores.^15^ This study shows that new chemical knowledge can be found from the hidden neurons of DNN. Backpropagation-based methods have also been used in DL toxicity models to extract important substructures for toxicity prediction and further identify potential toxicophores.^127,128^ Some software tools have been developed to facilitate DL interpretations. For example, Lucid (https://github.com/tensorflow/lucid, accessed January 2023) is an open-source toolbox containing methods for visualizing and interpreting neurons in ANN. iNNvestigate is a comprehensive library for implementing multiple interpretation methods for ANN models.^129^

### 3.3 Unsupervised Approaches

Besides the supervised learning algorithms discussed above, unsupervised techniques, such as clustering and matrix factorization algorithms, have been applied to study the feature variable relationships and reveal novel patterns. Clustering is the task of grouping a set of objects so that objects in the same cluster are more similar to each other than those in other clusters (Figure 3C). Clustering methods have been applied to cluster gene expression profiles,^130^ biological assays,^131,132^ and chemicals^67,133^ in groups to help mechanistic interpretations and predictions of chemical toxicities. On the basis of the hypothesis that similar chemical share similar toxicological profiles, the read-across strategy was developed to predict toxicity for new compounds using similar compounds with known toxicity results, which is easy to interpret and implement.^134^ Traditional read-across studies are only based on chemical structure similarity.^135−137^ Software tools like ToxMatch and OECD QSAR Toolbox use chemical structure-based similarity to perform chemical grouping and read-across.^138,139^ Similar to QSAR, only using structural information is error-prone when compounds with similar structures exhibit dissimilar toxicities.^55^ To address this issue, a hybrid read-across strategy was developed by combining chemical structure similarity and biosimilarity, which is calculated on the basis of biological profiles (e.g., HTS assays, omics data).^55,140^ The hybrid read-across could improve the discriminational power to distinguish compounds with similar chemical structures and could reveal the potential toxicity mechanisms by examining the bioprofiles.^73,131,141^

Matrix factorization (MF) is a collaborative filtering algorithm commonly used in recommendation systems.^142^ It works by decomposing a high-dimensional matrix into a product of two low-dimensional matrices to capture key patterns in the data (Figure 3E). For example, high-dimensional biological data can be stored in a matrix with the feature values in rows and individual samples in columns. MF can be applied to characterize both features and samples by vectors of latent factors inferred from the original matrix.^72,143^ In a study of toxicogenomic modeling, an extended MF technique, group factor analysis (GFA) (Figure 3F), was applied to model the relationships between the drug–gene matrix and drug-toxicity matrix. The identified shared components could capture cross-expression and toxicity relationships, which represent molecular mechanisms of toxicity.^72^

## 4 Model-Agnostic Interpretation Methods for IML

Besides interpreting ML models on the basis of specific algorithms, some universal interpretation strategies can be applied after model training and treat a model as a black box without inspecting internal model parameters (i.e., model-agnostic) (Figure 2C).^24,36,90^

### 4.1 Perturbation-Based Explanation

Perturbation-based strategy modifies or removes parts of feature data to measure the corresponding change of the model output (Figure 2C). This method provides explanations in the form of feature contributions. Commonly used perturbation-based methods include sensitivity analysis and feature effect plots. Sensitivity analysis (SA) studies the correlation between the uncertainty in the model outputs and the uncertainty in the inputs.^144,145^ SA can be performed with perturbation made to remove/permute one or more features at a time. One simple approach is altering one-feature-at-a-time (OAT) to see changes of the outputs.^146^ However, the OAT approach does not fully explore the input space since it does not detect the interactions between input features. The variance-based method quantifies the contributions of input features to the variance of the model predictions by treating the input and output uncertainties as probability distributions.^145,147^ As a measure of sensitivity, the total effect index gives the total variance in output *Y* caused by a feature *X* and its interactions with any other input features, which allows full explorations of the input space accounting for interactions. In a Bayesian network model to predict chemical modes of action (MoA) for aquatic toxicology, SA is applied to examine individual and multiple features’ abilities to maximize MoA probabilities.^148^ Only highly influential features were used to predict MoA, which reduced the model complexity and aided model interpretations. Feature effect plots is a powerful interpretation tool that visualizes the effects of features on the model’s outputs. The partial dependence plot (PDP) visualizes the average partial dependence between the predicted label and one or two features while keeping all other features fixed.^91^ It can show whether the relationship between the prediction and a feature is linear, monotonic, or more complex. For example, in modeling chemicals’ P-glycoprotein (P-gp) transport, Svetnik et al. selected 49 descriptors with high feature importance, which were related to functional groups, to do PDP visualization.^149^ Trends in PDPs can indicate whether a functional group tends to raise or lower P-gp activity and infer potential structure–activity relationships. An individual conditional expectation (ICE) plot extends PDP and visualizes the dependence of predictions on one or two features for each instance separately, thereby resulting in one curve per instance.^150−152^ ICEs can reveal individual differences and identify subgroups and interactions between model inputs. Goldstein et al. demonstrated the use of ICE plots to analyze how different subjects respond to depression treatments in a clinical trial.^150^ A black box model was built to predict treatment response scores using 37 personal features of the subjects and one binary treatment-type descriptor (cognitive therapy as 0 and paroxetine as 1). An ICE plot of two features, marital status and treatment type, showed that cognitive therapy is generally predicted to do better with married subjects, and paroxetine is predicted to do better with unmarried subjects. PDP and ICE plots are easy to interpret; however, they may miss important features since the partial dependence of the examined features (up to two) is computed on the basis of the assumption that they are not correlated with other features.^91^ Several software tools have been developed to facilitate perturbation-based interpretations. For example, the *iml* R package implements many model-agnostic methods, including PDP, ICE, and feature importance.^153^ The *ICEbox* and *pdp* R packages can be used for making ICE and PDP plots, respectively.^150,154^ In addition, several software tools have been developed to perform sensitivity analysis, including the *sensitivity* R package (https://cran.r-project.org/web/packages/sensitivity/index.html, accessed March 2023), the *SAlib* python library,^155^ and the *SAFE* (Sensitivity Analysis For Everybody) MATLAB package.^156^

### 4.2 Approximation-Based Explanation

Approximation-based methods involve learning an interpretable model (i.e., a surrogate model) to approximate the output of a black box model (Figure 2C). Training a surrogate model does not require information about the inner structure of the black box model, but access to the input data and model output is sufficient.^91^ With an input data set and its corresponding output from a black box model, an interpretable surrogate model can be trained. The performance of a surrogate model can be measured in approximating predictions of the black box model and interpreting the surrogate model. Examples of surrogate models include linear models for characterizing linear relationships and decision trees and decision rules for characterizing nonlinear relationships.^157,158^ A limitation of surrogate models is that complex black box models cannot be well approximated. To address this, an approach known as local interpretable model-agnostic explanations (LIME) was developed to focus on a small subset of instances.^159^ LIME starts with instances of interest to generate a new data set consisting of perturbed features and the corresponding output of a black box model. Then, LIME trains an interpretable linear model, which is a good approximation of predictions for the instances of interest. The predictions of the black box model can be explained by examining the parameters of the linear model. For example, in a DL modeling study for *in vitro* toxicity predictions, Ramsundar et al. applied LIME to extract potential toxicophores responsible for relevant toxicity.^160^ Sometimes, a linear surrogate model could lead to poor performance when the local relationship is nonlinear. Another local approximation-based approach, *anchors*, has been developed to characterize nonlinear relationships using decision rules.^161^ Anchor explanations are effective in capturing nonlinear behaviors and can highlight the part of feature data that is sufficient for making a prediction. The *anchors* method was implemented in the python package *anchor*,^161^ and integrated in *alibi*,^162^ a Python library for ML model inspection and interpretation.

## 5 Toxicological Knowledge in Guiding the Design of IML Models

Although posthoc interpretations are useful in understanding important features affecting toxicity predictions, they can be unreliable and misleading when the model is not appropriately designed. If self-explanatory models incorporate interpretability directly to the model structures, they can provide explanations to what the model computes.^27,90^ For toxicity evaluations, an IML model can be developed to follow the toxicology knowledge (Figure 2B). In the past decade, systemic understanding of chemical toxicity in organisms using knowledge from an AOP framework and systems toxicology has become a trend.^163−165^ The knowledge base frameworks represent a sequence and/or network of ordered events leading to adverse outcomes that show the interactions between toxicity-related components that can guide the design of intrinsic IML models in toxicity predictions.

### 5.1 The AOP Framework

An AOP is a structured representation of linked events between a molecular initiating event (MIE) (e.g., molecular interaction between a chemical and a receptor) and an adverse outcome in organisms (Figure 2B).^59,166^ The MIE triggers a cascade of key events that occur at different biological levels relevant to adverse outcomes. The AOP-Wiki (aopwiki.org) is the primary repository for international AOP development efforts coordinated by the Organisation for Economic Co-operation and Development (OECD) (Table 2). Currently, the AOP-Wiki features more than 400 AOPs, which include those for various toxicity endpoints, such as acute inhalation toxicity,^167^ reproductive and developmental toxicity,^168^ and cholestatic and steatosis liver injury.^169^ The AOP development efforts, together with publicly available toxicity big data, pave the way for computational AOP modeling that is more interpretable than traditional ML models. Moreover, the AOP structure organizations can be applied in designing IML models for efficient toxicity predictions. By integrating the chemical structure data and results of mechanism-based assays characterizing key events in AOP, the pathway models can systematically assess the potential of a compound to induce the target adverse outcome.^170^

An individual AOP may focus on a specific pathway, where mechanistically linked events proceed to a toxicity effect in a unidirectional and linear way.^163,171^ In a mechanistic model for hepatotoxicity predictions, the toxicity potential of a chemical is assessed on the basis of whether it (1) possesses certain structural alerts and (2) activates the antioxidant response element (ARE) pathway, an oxidative stress-related key event.^172^ This is a decision rule-based model, where chemicals that satisfy both two conditions are predicted as toxic, and chemicals that dissatisfy both two conditions are predicted as nontoxic. Chemicals that possess the identified structural alerts are suspected to be metabolized into reactive intermediates (i.e., MIE), which can trigger oxidative stress in the liver, thereby forming a plausible pathway that leads to hepatotoxicity. The limitation of this model is that it only can evaluate a small portion of hepatotoxicants since other toxicity mechanisms besides oxidative stress can also lead to hepatotoxicity. After the inclusion of additional assays representing key events in other AOPs, some false negative predictions could be corrected.

Several AOPs sharing at least one common component can form an AOP network.^163,171,173^ They can focus on a single adverse outcome but describe different MIEs leading to this adverse outcome or share the same MIE but diverge to different AOPs.^173^ Judson et al. reported a network model for chemical perturbations of the estrogen receptor (ER).^174^ This model integrated three associated pathways, ER agonist, ER antagonist, and pseudoreceptor pathways, and utilized data from 18 HTS bioassays to identify ER agonists and antagonists. In a recent study, a knowledge base DNN model was developed to mimic the toxicity pathway for ER agonists using a virtual AOP framework.^175^ In the DNN architecture, 57 HTS bioassays were organized among five network layers on the basis of the biological processes in the ER pathway—receptor binding as MIE, receptor dimerization, DNA binding, transcriptional activation, and cell proliferation as key events—and eventually led to the adverse outcome of *in vivo* rodent uterotrophic bioactivity. This model could efficiently and accurately evaluate rodent uterotrophic bioactivity for new compounds [area under the receiver operating characteristic (ROC) curve (AUC) = 0.864–0.927], which outperformed the QSAR model that only used chemical structure data as inputs (AUC = 0.594). Moreover, the model could virtually simulate the perturbations in the toxicity pathway for each predicted toxic compound. The design of DNN models to mimic AOP networks is promising for developing future interpretable models of complicated toxicity endpoints.

### 5.2 Systems Toxicology

AOPs are usually constructed by literature compilations and focus on the states of a series of systems (cells, tissues, organs, organisms), whereas systems biology studies the molecular details (e.g., genes, proteins, metabolites) of these biological systems using -omics technologies.^163,176^ As part of systems biology, systems toxicology describes the toxicological evaluation of biological systems, which involves perturbating systems by toxicants and stressors, monitoring molecular expressions and conventional toxicological parameters, and iteratively integrating response data.^71,177^ Toxicology programs, such as ToxCast/Tox21 and L1000, have made progress in integrating data from diverse technologies and endpoints in different levels into systems biology approaches for toxicity evaluations.^62,81^ Knowledge databases that reflect the functional characterization of components and interactions among diverse components provide informatic tools to support systems toxicology (Table 2). For example, the Gene Ontology database annotates each gene product regarding molecular function, biological process, and cell component, and relationships among these annotations form a loose hierarchical network (Figure 2B).^178,179^ Reactome is a pathway database where relations of signaling and metabolic molecules are organized into a hierarchical network of biological pathways and processes (Figure 2B).^180^ These structural knowledge bases enable the ML modeling of a biological system from the molecular level to larger pathways and cellular and even organism-level systems.

A recent development of a visible neural network (VNN) reported the integration of ontological and pathway hierarchy in the design of interpretable DNN models.^181−185^ In VNNs, the connectivity of neurons in different layers is set to mirror the biological hierarchy. Genes or proteins as inputs only connect to specific neurons representing their associated pathways, and these pathway neurons subsequently connect to their parent pathways, thereby making it a sparse DNN with reduced complexity and intrinsic interpretability. Kuenzi et al. developed a VNN model named DrugCell to simulate the response of human cancer cells to therapeutic compounds.^182^ DrugCell was designed with two parts: a VNN modeling the hierarchical organization of Gene Ontology terms and a conventional ANN embedding the chemical fingerprints. DrugCell could correctly predict drug responses (Spearman correlation rho = 0.8 between predicted and observed response value), which significantly outperformed the elastic net regression model (*p* < 0.0001). Furthermore, DrugCell could provide insights into the underlying mechanisms of action by inspecting the simulated pathway neuron states. Elmarakeby et al. developed a VNN model named P-NET that integrates Reactome hierarchical knowledge to predict cancer state on the basis of patients’ genomic profiles.^184^ The trained P-NET model outperformed classic ML models, including SVM, logistic regression, and decision trees, with AUC = 0.93 and accuracy = 0.83. Additionally, P-NET demonstrated significantly better performance than the traditional dense DNN model in sample sizes up to 500 (*p* < 0.05). In a recent study, Hao et al. reported a VNN model named DTox (deep learning for toxicology) for predict a chemical’s outcomes in 15 toxicity assays.^183^ DTox connects target protein profiles (input features) to toxicity assay outcomes (outputs) by hidden neurons mapping to the Reactome pathways. DTox can achieve the same level of performance as classic ML approaches and further explain toxicity mechanisms by identifying VNN paths. Since the gene expression change of the components constituting a pathway may reflect whether the pathway is perturbed, the identified VNN paths were further validated by differential expression analysis using LINCS transcriptomic profiles.

## 6 Implications and Perspectives

In toxicology, ML-based computational modeling is a promising alternative method to traditional animal models for predicting chemical toxicity potentials. In the current big data era, chemical toxicity data continue to grow at a rapid pace, and advanced ML and DL approaches are urgently needed to deal with these data. Interpretability is critical for the application of ML in risk assessments of chemicals that may impact human and environmental health. In the above sections, we have presented strategies for applying IML in computational toxicology, including the use of interpretable feature data, interpretation methods, and the development of intrinsic IML models using knowledgebase frameworks in toxicology.

Data standardization and curation are critical in computational modeling approaches, where care should be taken to avoid introducing technical artifacts and to ensure the quality of modeling sets, as well as the resulting model performance and interpretation.^196^ Many modeling studies use well-established molecular representations as features, such as properties, binary fingerprints, and geometrical descriptors, which can capture chemical and structural features defined in advance.^197,198^ The generation of these molecular representations is standardized, and various structure curation protocols are available to facilitate the chemical descriptor generations.^199,200^ However, as described above, toxicogenomic and assay data, which can capture intricate biological responses for chemicals of interest, are much more diverse, heterogeneous, and unstandardized than chemical structures. Experimental conditions and protocols for generating these data can vary widely among laboratories, thereby leading to poor data quality in some data resources that may impact model performance and interpretability.^7^ Similarly, assay data from various studies and HTS programs may exhibit different data structures (e.g., classifications, dose/concentration dependent curves, or even raw data) and may have inconsistent results for the same chemicals.^8,201^ When collecting training data from multiple resources, curation workflows, such as those employed by Integrated Chemical Environment and ChEMBL, should be implemented to ensure data quality and integrity.^86,202,203^

The interpretation strategy should be tailed to what needs to be learned from the model.^196,204^ Traditionally, understanding which structure features of a toxicant contribute to its toxicity is useful to toxicologists for decision making and medicinal chemists to modify the molecule.^109,205^ Mechanistic explanations of the model predictions are crucial for high-stakes decision-making, such as determining whether a chemical is safe for humans and the environment. However, there are still many challenges to be resolved, and further efforts are needed to advance IML in this field. For example, new mechanistic IML models are trained on heterogeneous data (e.g., chemical structure, gene expression, and bioactivities), which increases the complexity of modeling tasks and makes it challenging to identify critical features and explain underlying mechanisms. Another issue caused by such heterogeneous data is the existence of missing values in the feature profiles for target compounds, which is a common issue in big data modeling.^5,206^ Methods to impute missing values (e.g., read-across) may introduce uncertainties in the training data and the following modeling procedure. Therefore, the development of novel and interpretable representations of chemicals will be critical in future research.^204^ Intrinsic interpretable models that incorporate toxicological knowledge frameworks can overcome the challenges posed by big data by providing both mechanism explanations and accurate predictions.^204^ In QSAR modeling, the chemical descriptor space is normally used to define the applicability domain of the model to assess whether the prediction for a target chemical is reliable or not.^207−209^ However, it is difficult to define the applicability domain for mechanistic models, especially those using DL and heterogeneous data. In terms of IML explanations, a challenge lies in how to measure the levels of interpretability, compare the interpretability of different IML models, or determine the faithfulness of different interpretation methods to the same model.^40,90^ There are no universal criteria for selecting ML approaches for toxicological modeling, nor is there a clear choice for the optimal interpretation methods. Confidence in the interpretation results will be enhanced when multiple approaches yield consistent conclusions.^196^ As many interpretation strategies being developed for IML in the toxicology community, the use of these strategies can require significant computational knowledge for toxicologists. Further development and improvements of user-friendly software platforms can facilitate the design, validation, and acceptance of IML and its associated explanations.
